# Pathogenesis and shedding of Usutu virus in juvenile chickens

**DOI:** 10.1080/22221751.2021.1908850

**Published:** 2021-04-09

**Authors:** Sarah C. Kuchinsky, Francesca Frere, Nora Heitzman-Breen, Jacob Golden, Ana Vázquez, Christa F. Honaker, Paul B. Siegel, Stanca M. Ciupe, Tanya LeRoith, Nisha K. Duggal

**Affiliations:** a Department of Biomedical Sciences and Pathobiology, Virginia-Maryland College of Veterinary Medicine, Virginia Polytechnic Institute and State University, Blacksburg, VA, USA; b Department of Mathematics, Virginia Polytechnic Institute and State University, Blacksburg, VA, USA; c Department of Biological Sciences, Virginia Polytechnic Institute and State University, Blacksburg, VA, USA; d National Centre for Microbiology, Instituto de Salud Carlos III (ISCIII), Epidemiology and Public Health Network of Biomedical Research Centre (CIBERESP), Madrid, Spain; e Department of Animal and Poultry Sciences, Virginia Polytechnic Institute and State University, Blacksburg, VA, USA

**Keywords:** Usutu virus, pathogenesis, avian model, juvenile chicken, virus-host interactions

## Abstract

Usutu virus (USUV; family: *Flaviviridae,* genus: *Flavivirus*), is an emerging zoonotic arbovirus that causes severe neuroinvasive disease in humans and has been implicated in the loss of breeding bird populations in Europe. USUV is maintained in an enzootic cycle between ornithophilic mosquitos and wild birds. As a member of the Japanese encephalitis serocomplex, USUV is closely related to West Nile virus (WNV) and St. Louis encephalitis virus (SLEV), both neuroinvasive arboviruses endemic in wild bird populations in the United States. An avian model for USUV is essential to understanding zoonotic transmission. Here we describe the first avian models of USUV infection with the development of viremia. Juvenile commercial ISA Brown chickens were susceptible to infection by multiple USUV strains with evidence of cardiac lesions. Juvenile chickens from two chicken lines selected for high (HAS) or low (LAS) antibody production against sheep red blood cells showed markedly different responses to USUV infection. Morbidity and mortality were observed in the LAS chickens, but not HAS chickens. LAS chickens had significantly higher viral titers in blood and other tissues, as well as oral secretions, and significantly lower development of neutralizing antibody responses compared to HAS chickens. Mathematical modelling of virus-host interactions showed that the viral clearance rate is a stronger mitigating factor for USUV viremia than neutralizing antibody response in this avian model. These chicken models provide a tool for further understanding USUV pathogenesis in birds and evaluating transmission dynamics between avian hosts and mosquito vectors.

## Introduction

As an emerging zoonotic virus, Usutu virus (USUV, family: *Flaviviridae,* genus: *Flavivirus*) is an increasingly important global public and wildlife health concern [1]. USUV was first isolated from a *Culex neavei* mosquito in South Africa in 1959 and has since been detected throughout sub-Saharan Africa, central Europe, and the Mediterranean Basin [2,3]. USUV belongs to the Japanese encephalitis serocomplex, where it shares similar antigenic properties with West Nile virus (WNV) and St. Louis encephalitis virus (SLEV), both neuroinvasive arboviruses found in wild bird populations in the United States [4]. USUV is maintained in an enzootic cycle between ornithophilic mosquitos, primarily *Culex* spp., and wild birds [5–7]. Incidental “spillover” infections in mammals, including humans, are also known to occur [4,8–11]. To date, 79 USUV infections have been reported in humans, with symptoms ranging from asymptomatic or mild febrile illness, to meningoencephalitis or encephalitis [4,12–17].

The earliest indication of USUV circulating in European wild bird populations occurred in 2001 in Austria [18], though retrospective analysis suggests that USUV was circulating five years earlier in Italy [19]. Since its establishment in Europe, USUV has been implicated in a decline of breeding bird populations [20,21]. The burden of disease in wild bird populations has been most prominent in the Eurasian blackbird (*Turdus merula*) [20,22], great grey owl (*Strix nebulosa*) [21], and house sparrow (*Passer domesticus*) [23].

While several murine models have been developed to evaluate human USUV pathogenesis [24–28], the use of an avian model is essential to understanding the characteristics of transmission dynamics and avian pathogenesis. Experimental inoculation attempts with USUV have failed to reproduce infection (as evidenced by virus isolation) in two domestic avian hosts. Two-week-old domestic geese (*Anser anser domesticus*) inoculated intramuscularly with USUV strain 939/01 (Vienna 2001) were positive for USUV nucleic acid in pooled organ samples, a pharyngeal swab, and a single plasma sample by RT-PCR [29]. Viral nucleic acid was detected in two-week-old chickens intravenously inoculated with USUV strain 939/01 (Vienna 2001) in pooled organs, cloacal and pharyngeal swabs, and isolated PBMCs [30]. Although these *in vivo* studies reveal that different domestic avian species are susceptible to USUV, neither resulted in individuals developing infectious virus in blood, which is critical for transmission to mosquitos. Viral RNA in serum was observed in domestic canaries (*Serinus canaria*) inoculated intraperitoneally with USUV strain UR-10-Tm (Italy 2010); viral RNA was also detected in organs, droppings, and feathers of 3 inoculated canaries [31]. Thus, an avian model of USUV infection with infectious virus has not yet been reported.

The domestic chicken (*Gallus gallus domesticus*) has commonly been used as a model of infection and surveillance measures in flavivirus infections, including Murray Valley encephalitis virus [32,33], SLEV [34,35], WNV [36,37], and USUV [38]. Although viremia has been observed in WNV experimentally infected chickens, there appears to be an age dependent difference in viral titer, where an adult chicken model reaches peak viremia of 4 log_10_ PFU/mL [36] and a two-day-old chick peaks at 7 log_10 _PFU/mL [37]. Age-related differences in viremia levels have also been observed in one-day-old compared to one-week-old SPF chickens inoculated with WNV, with detectable viremia on days 2–7 post-inoculation in one-day old chickens and only on day 2 post-inoculation in one-week old chickens [39]. These studies suggest that age of infection is crucial for a flavivirus avian model and that a juvenile chicken model could be employed to discern the viral kinetics of USUV.

The primary goal of this study was to develop a model of USUV in birds that could be used to study viral pathogenesis and transmission. Juvenile commercial ISA Brown chickens and two chicken lines that were previously bred from a common founder population for high (HAS) or low (LAS) antibody response to sheep red blood cells [40–42] were experimentally inoculated with USUV. We found the LAS chickens developed clinical disease, as well as significantly higher viremia compared to HAS chickens. Mathematical modelling showed that the viral clearance rate is a stronger mitigating factor for altering USUV viremia than neutralizing antibody responses. These models provide a tool for further evaluating avian-mosquito transmission dynamics of USUV.

## Materials and methods

### Virus isolates

USUV isolates used throughout the study were: HU10279-09 (Spain 2009, Africa 2 lineage, MN813489, passage 2+, isolated from *Culex perexiguus*) [43], TMNetherlands (Netherlands 2016, Europe 3 lineage, MN813490, passage 5, isolated from *Turdus merula*) [21], UG09615 (Uganda 2012, Africa 3 lineage, MN813491, passage 3, isolated from *Culex sp.*) [44], and SAAR1776 (South Africa 1959, Africa 2 lineage, MN813492, passage 9, isolated from *Culex neavei*) [3]. These strains have been previously sequenced by our lab [28].

### Cell lines

Vero cells were grown at 37°C with 5% CO_2_ and maintained in Dulbecco’s Modified Eagle’s Medium (DMEM) (Fisher Scientific), supplemented with 5% Fetal Bovine Serum (FBS) and 1% penicillin–streptomycin. Cells were plated at 6.5 × 10^4^ cells/well in 12-well plates for plaque assays or 1.65 × 10^5^ cells/well in 6-well plates for PRNTs.

DF-1 chicken fibroblast cells were grown at 37°C with 5% CO_2_ and maintained in Dulbecco’s Modified Eagle’s Medium (DMEM) (Fisher Scientific), supplemented with 10% Fetal Bovine Serum (FBS) and 1% penicillin–streptomycin. Cells were plated at 1.8 × 10^5^ cells/well in 12-well plates and inoculated one day later with USUV strain at a multiplicity of infection (MOI) of 0.1 in triplicate. Serial timepoints were collected every 24 h for five days. This was repeated twice for a total of three replicates. Viral titer was quantified by Vero cell plaque assay.

### Chicken experiments

#### USUV inoculation in ISA Brown chickens

Seventy ISA Brown mixed-sex chickens were obtained from the Poultry Research Center (Virginia Tech, Blacksburg, VA) and randomly assigned to isolator cages in an ABSL-2 facility. Six to ten birds were housed in each isolator cage. Chickens acclimated for one day prior to inoculation. This experiment was split over two experimental sessions.

Groups of two-day-old chickens (*n *= 16) were subcutaneously inoculated in the abdominal region with 1500 PFU (50 μl inoculum) of one of four USUV strains (HU10279-09, TMNetherlands, UG09615, or SAAR1776) diluted in BA-1 viral transport media. Six 2-day-old chickens served as a control group and were injected with 50 μl of PBS.

For the high dose experiment, 24 ISA Brown mixed-sex chickens were inoculated as for the low dose group, except groups (*n *= 12) were subcutaneously inoculated with 10^5^ PFU (50 μl inoculum) of USUV strains.

A 50 μl blood sample was collected daily from either the jugular vein or brachial wing vein and stored in a serum-separator tube. Fluids (0.25–0.5 mL) were provided orally to each bird following venipuncture. Whole blood samples were centrifuged to separate serum and subsequently stored at −80°C until further processing. A final 100 μl blood sample was collected from 9 birds in each group on dpi 14. On days 3 and 5 post inoculation, a subset of 3 birds from each group was euthanized via CO_2_ asphyxiation followed by cervical dislocation. The following tissues were aseptically collected for viral titration and histopathology: brain, bursa, heart, kidney, liver, lungs, and spleen. Tissues were stored dry at −80°C for later quantification via plaque assay. Prior to freezing, a portion of each tissue was fixed in 10% neutral buffered formalin for histopathology. Oral and cloacal swab samples were also collected from euthanized individuals. Cotton-tipped swabs were premoistened in 0.5–1 mL BA-1 viral transport medium, and oral and cloacal samples were collected by swabbing the mucosal surfaces of the oropharynx and cloaca, respectively. The swab was gently placed inside the cavity and 2–4 circular passes against the mucosal surfaces were made. Efforts to keep fecal residue out of the transport medium were made [45]. Swab samples were stored at −80°C until further processing.

#### USUV inoculation in HAS and LAS chickens

The HAS and LAS chicken lines originated from a common White Leghorn founder population and have been bred for >40 generations for a single trait: high (HAS) or low (LAS) antibody response against sheep red blood cells [40,41]. Twenty-five chickens from each line were randomized into groups. Groups (*n *= 16) of one-day-old HAS and LAS chickens were subcutaneously inoculated with 1500 PFU (50 μl inoculum) of the Netherlands 2016 USUV strain. Nine 1-day-old chickens from each line served as a control group and were inoculated with 50 μl of PBS. Ten inoculated individuals from each line were bled daily for seven days, as described previously. A final blood sample was collected on dpi 14. On days 3 and 5 post inoculation, 6 birds from each line were euthanized via CO_2_ asphyxiation followed by cervical dislocation. Tissues and oral and cloacal swabs were collected from euthanized individuals and processed as described previously. If clinical signs including lethargy, ruffled feathers, poor responsiveness, or weight loss ≥ 15% were observed, then birds were euthanized via CO_2_ inhalation, followed by cervical dislocation.

All experiments were performed in accordance with the Virginia Tech Institutional Animal Care and Use Committee (IACUC #18-069). Throughout the experiments, commercial feed and fresh water were provided *ad libitum*. Chickens were monitored daily for clinical signs by animal care staff and research personnel.

### Viral quantification assays

Viral titers of serum, tissues, and oral and cloacal swabs were quantified through Vero cell plaque assay. Tissues were weighed and suspended in equal parts BA-1 medium, then homogenized through bead homogenization in a Qiagen TissueLyserLT at 50 oscillations/sec for 2–6 min. Samples were clarified by centrifugation at 18,500 rpm for 3 min. The limits of detection were 2 log_10 _PFU/mL for serum samples, 1.7 log_10 _PFU/g or 0.3 log_10 _PFU/tissue for tissue samples, and 0.4 log_10 _PFU/swab for oral and cloacal secretions.

### PRNT assays

Blood collected at 14 days post inoculation was assayed by plaque reduction neutralization test (PRNT). Sera were heat inactivated at 56°C for 30 min and incubated with approximately 1000 PFU of the homologous USUV strain for 1 h at 37°C before plating on Vero cells. Neutralization activity was defined by plaque reduction at a 90% threshold [46].

### Histopathology

Following euthanasia, tissues were collected and stored in 10% neutral buffered formalin prior to standard processing and paraffin embedding. Sections were cut at 5 μm and stained by routine hematoxylin–eosin (H&E) staining for histopathological analysis. Tissues were semi-quantitively evaluated for the number of foci of inflammation; scores were summed for a composite pathology score. The number of foci of inflammation in 5, 200× fields was counted. A composite score scale is as follows 0 = 0; 1 = 1–5 foci; 2 = 6–10 foci; 3 = 11–15 foci; 4 = 16 + . The median pathology score was use for comparison of lesions across groups. Slides were analysed by a board-certified veterinary pathologist.

### Mathematical modeling of virus-host interaction

A target cell-limited model (Equation 1) was used to determine the kinetics of the virus titers in USUV-infected HAS and LAS chickens. The model is described by the following variables. Leukocytes susceptible to USUV, called target cells (T), become infected at constant rate β mL per virion per day resulting in latently infected cells (E). Latently infected cells become productively infected cells (I) at rate k per day. Productively infected cells produce virus at rate p per infected cell per day and are removed at rate δ per day. The removal rate accounts for both natural death and the possibility that the antibody is enhancing the rate of infected cell loss through antibody-dependent cellular cytotoxicity or complemented mediated lysis. Virus (V) is removed at rate c per day. The removal rate accounts for both virus degradation and the possibility of antibody enhancing the rate of virus clearance through opsonization. The initial target cell concentration is estimated to be $T_{0}=10,000$ leukocytes/ml. The viral inoculum is $V_{0}=1500$ PFU which, when distributed through 2.5 mL of blood, gives an initial $\log_{10}$ virus concentration of $\log_{10}V_{0}=2.78$ PFU/ml. There were no infected cells at the beginning of the study, therefore, we set $E_{0}=I_{0}=0$ leukocytes/ml.

(1) $$\begin{array}{rl} & \frac{dT}{dt}=-\text{β}TV\\ & \frac{dE}{dt}=\text{β}TV-kE\\ & \frac{dI}{dt}=kE-\delta I\\ & \frac{dV}{dt}=pI-cV \end{array}$$

#### Parameter estimation

Data fitting was performed by comparing HAS and LAS virus titer data at times $t_{i}\log_{10}D_{i}$ to predicted virus population at times $t_{i}$ as given by model (Equation 1) $\log_{10}V(t_{i})$, for i=1,2,…n. Initially, the HAS and LAS groups were considered to have similar characteristics. A non-linear mixed effects model and the stochastic approximation expectation-maximization (SAEM) algorithm implemented in Monolix Suite 2019R1 (Lixoft 2019), with log normally distributed parameters, point one parameter variation, and proportional residual errors were used to minimize the functional $J(param)=\underset{param}{\min}{\left(\sum_{k=1}^{T_{H}+T_{L}}\frac{1}{T_{H}+T_{L}}{\sum_{i=1}^{n}\left(\log_{10}D_{i}^{k}-\log_{10}V^{k}(t_{i},\;param)\right)}^{2}\right)}^{1/2}$, where param={k,p,β,δ,c}, $T_{H}=13$ is the number of subjects in the HAS group, $T_{L}=13$ is the number of subjects in the LAS group, and n is the number of data points in each subject. These initial estimates were used to assign values to two parameters, k and p to avoid non-identifiability. The remaining parameters $param_{r}=\{\text{β},\;\delta ,\;c\}$ were then assumed to be HAS or LAS population specific. They were estimated by performing HAS only and LAS only population fits using a non-linear mixed effect model as well as individual fits for each subject in the HAS and LAS groups, using the method and specification described above.

Additionally, the reproduction number (or basic reproductive ratio), defined as the number of infected cells (or virus particles) that are produced by one infected cell (or virus particle), when the virus is introduced into a population of uninfected target cells $T_{0}$ was estimated for HAS and LAS populations. It is given by $R_{0}=\frac{\text{β}p}{c\delta }T_{0}$.

### Statistical analysis

Descriptive statistics including means and standard deviations of viremia, viral tissue titers, and viral swab titers were calculated for chicken studies. Survival curves were analysed by Mantel–Cox test. Viremia and weight change data were analysed using Repeated Measures two-way ANOVA with Tukey’s or Sidak’s multiple comparisons tests, as appropriate. Tissue and swab data were analysed using two-way ANOVA with Tukey’s or Sidak’s multiple comparisons tests, as appropriate. Fisher’s exact test was used to compare the results of PRNT_90_ assays. Data were analysed and graphed using GraphPad QuickCalcs and GraphPad Prism 9 (GraphPad Software, San Diego, CA).

## Results

### Recent USUV isolates replicate to higher titers compared to the prototypic USUV isolate

**Figure 1.**
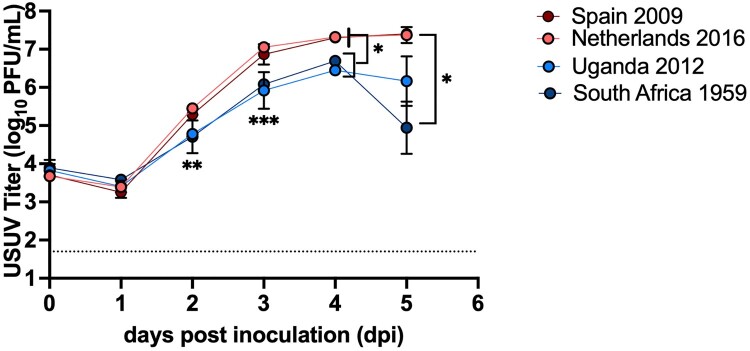
Growth kinetics of USUV isolates in DF-1 cells. The growth curve was performed three times, with one representative experiment shown. Symbols represent mean and error bars represent standard deviation of triplicate inoculated cultures. The limit of detection is indicated by dashed line. *p < 0.05, **p < 0.01, ***p < 0.001.

Growth kinetics of four USUV strains: Uganda 2012, South Africa 1959, Spain 2009, and Netherlands 2016 were evaluated *in vitro* in DF-1 chicken fibroblast cells. Uganda 2012 and South Africa 1959 had an average peak titer of 6.4 log_10 _PFU/mL and 6.7 log_10 _PFU/mL, respectively, on dpi 4. Spain 2009 and Netherlands 2016 reached an average peak titer of 7.4 log_10 _PFU/mL on dpi 5 (Figure 1). The prototypic strain, South Africa 1959, generated significantly lower titers compared to Netherlands 2016 on dpi 2–3 and 5 (Figure 1, *p *< 0.05). Both European strains generated significantly higher titers than both African strains on dpi 4 (Figure 1, *p *< 0.05).

### Two-day-old chickens develop viremia when inoculated with African and European strains of USUV

**Figure 2.**
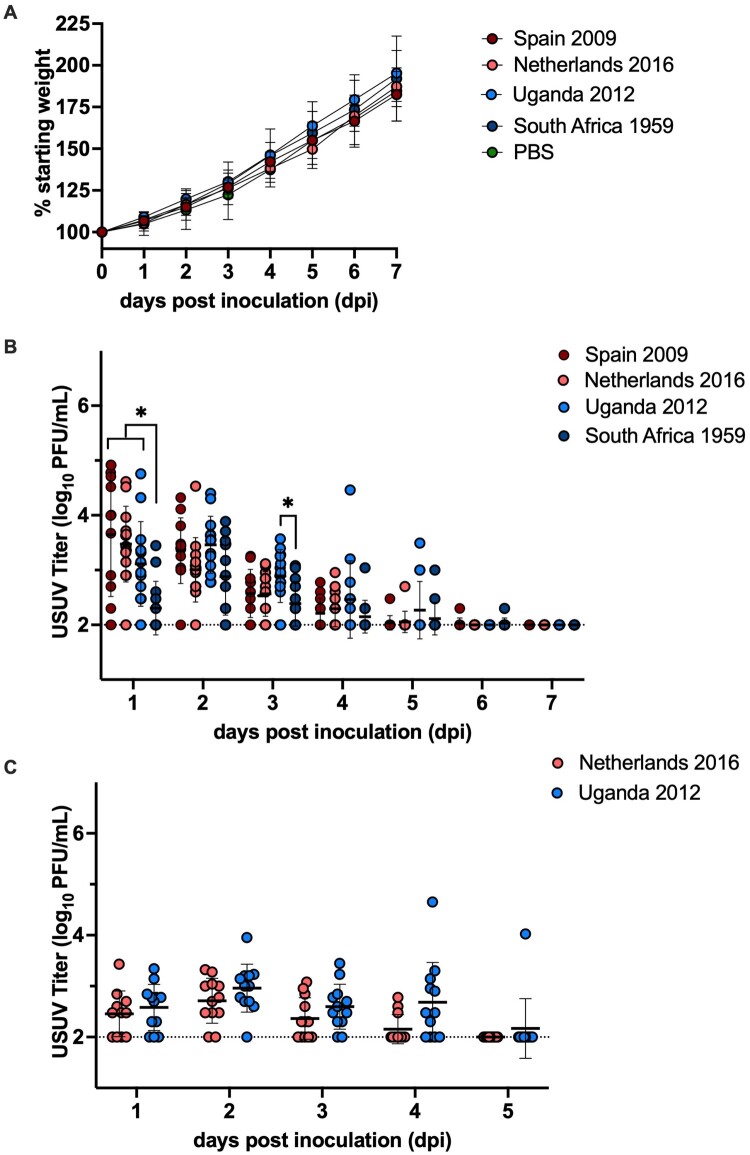
Serum titers of two-day-old ISA Brown chickens inoculated with African and European strains of USUV. (A) Mean percentage of initial starting weight of chickens following 1500 PFU USUV inoculation. (B) Serum titers of chickens inoculated with 1500 PFU of USUV. (C) Serum titers of chickens inoculated with 10^5^ PFU of USUV. Circles represent individual samples; lines represent mean; error bars represent standard deviation. The limit of detection is indicated by dashed line. *p < 0.05.

In order to evaluate USUV infection in an *in vivo* avian model, two-day-old ISA Brown chickens were subcutaneously inoculated with 1500 PFU of one of four USUV strains: Uganda 2012, South Africa 1959, Spain 2009, or Netherlands 2016. There was 100% survival across all inoculated subjects and no clinical signs of disease, including weight loss, were observed (Figure 2(A)). Of the 52 inoculated chickens bled consecutively, all but 1 became viremic. Chickens inoculated with the prototype strain, South Africa 1959, developed significantly lower serum titers compared to the three other USUV isolates on dpi 1 and compared to Uganda 2012 on dpi 3 (Figure 2(B), *p *< 0.05). The peak mean serum titer for Spain 2009 and Netherlands 2016 occurred on dpi 1 at 3.6 log_10 _PFU/mL and 3.5 log_10 _PFU/mL, respectively. The peak mean titer in serum for Uganda 2012 and South Africa 1959 occurred on dpi 2 at 3.5 log_10_ PFU/mL and 2.9 log_10 _PFU/mL. Chickens sustained detectable viremia for at least five days post inoculation, with one individual still viremic on dpi 6.

To determine whether a higher inoculum dose would increase viral titer in serum, two-day-old ISA Brown chickens were inoculated with 1 × 10^5^ PFU of either Netherlands 2016 or Uganda 2012. There was 100% survival, and all chickens developed viremia. The peak mean titer for Netherlands 2016 and Uganda 2012 occurred on dpi 2 at 2.7 log_10 _PFU/mL and 3.0 log_10 _PFU/mL dpi 2, respectively. There were no statistically significant differences between the two strains across all timepoints (Figure 2(B), *p *> 0.20). Mean titer was significantly lower in the Netherlands 2016 high dose group relative to its low dose counterpart on dpi 1 (*p *< 0.01). Together, these data indicate that two-day-old chickens are susceptible to infection by African and European USUV isolates.

### Viral dissemination and histopathology in heart tissue of USUV-inoculated chickens

**Figure 3.**
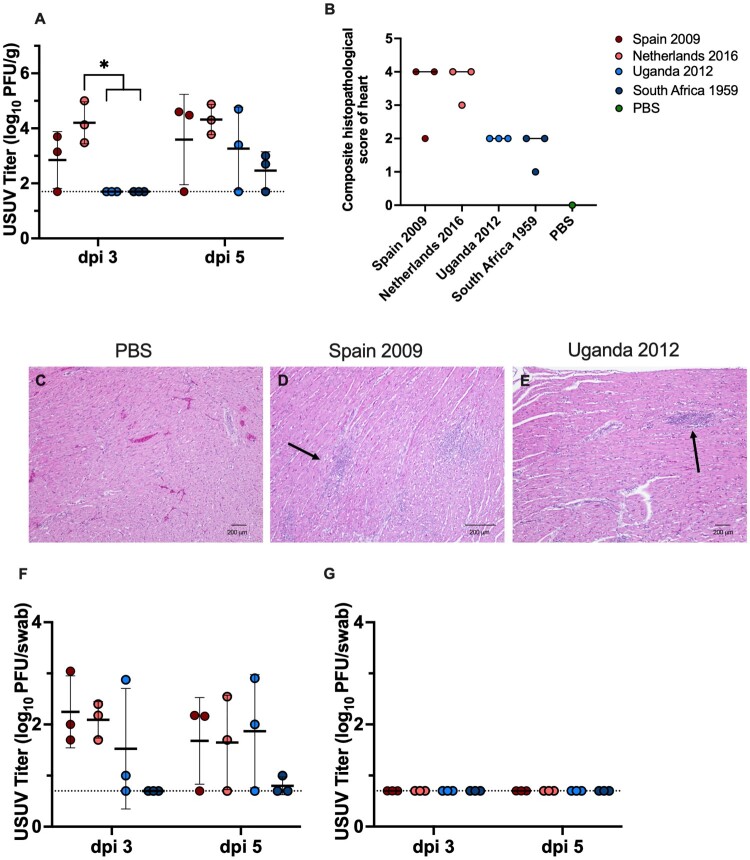
Evidence of USUV dissemination and USUV-mediated pathology in heart tissue from ISA Brown chickens inoculated with African and European strains of USUV. (A) Viral titer in heart tissue collected on dpi 3 and dpi 5. Circles represent individual samples; lines represent mean; error bars represent standard deviation. The limit of detection is indicated by dashed line. *p < 0.05. (B) Composite histopathology scores of heart tissue; lines represent median. (C) Representative image of heart tissue collected on dpi 5 from chickens inoculated PBS, with no inflammation (H&E stain). (D) Representative image of heart tissue collected on dpi 5 from chickens inoculated with European USUV strain, with foci of lymphocytic and heterophilic infiltrates (arrow) (H&E stain). (E) Representative image of heart tissue collected on dpi 5 from chickens inoculated with African USUV strain, with foci of inflammation (arrow) (H&E stain, scale bars = 200µm). (F) Viral titer in oral swabs collected on dpi 3 and dpi 5. (G) Viral titer in cloacal swabs collected on dpi 3 and dpi 5. Circles represent individual samples; lines represent mean; error bars represent standard deviation. The limit of detection is indicated by dashed line.

To assess for viral dissemination and evidence of disease, tissues were collected from three individuals per low-dose experimental group on dpi 3 and dpi 5. Infectious viral titer was quantified in tissue samples, and tissue sections were evaluated for histopathology. Infectious virus was isolated from heart samples of chickens inoculated with either of the European strains on dpi 3, with Netherlands 2016 group producing significantly higher viral titers than either of the African strains (Figure 3(A), *p *< 0.05). By dpi 5, infectious virus was isolated from heart tissue of chickens across all USUV isolates. Sections of heart collected on dpi 5 were also evaluated microscopically. Hearts were semi-quantitively evaluated for the number of foci of inflammation; scores were summed for a composite pathology score. Juvenile chickens infected with Netherlands 2016 or Spain 2009 had similar scores and were characterized by more foci of inflammation compared to the chickens inoculated with Uganda 2012 or South Africa 1959 (Figure 3(B)). No inflammation was observed in heart tissue collected from control chickens inoculated with PBS (Figure 3(C)). A representative image from a chicken infected with Spain 2009 with numerous foci of lymphocytic and heterophilic infiltrates is shown (Figure 3(D)). Representative images from a chicken infected with Uganda 2012 with limited inflammation (Figure 3(E)), and from sham-inoculated control bird with no inflammatory foci (Figure 3(C)) are shown. Additional tissues were collected, and infectious virus was isolated from at least one individual across all virus strains tested but evidence of disease was not observed histologically.

To assess viral shedding, oral and cloacal swabs were also collected from individuals euthanized for tissue collection. Infectious virus was detected in oral swabs of chickens inoculated with all virus strains on dpi 5 (Figure 3(F)). No infectious virus was detected in cloacal swabs collected from chickens inoculated with USUV at either time point (Figure 3(G)).

A PRNT_90_ was used to determine the development of neutralizing responses in serum on dpi 14 against homologous USUV strains. At least two samples from each experimental group generated a neutralizing response against USUV at a reciprocal PRNT_90_ titer of 20 (Supplemental Table 1). These data indicate that ISA Brown chickens generated a similar neutralizing response to African and European USUV strains.

### Statistically significant greater morbidity and viremia in chickens from a line selected for low antibody responses

**Figure 4.**
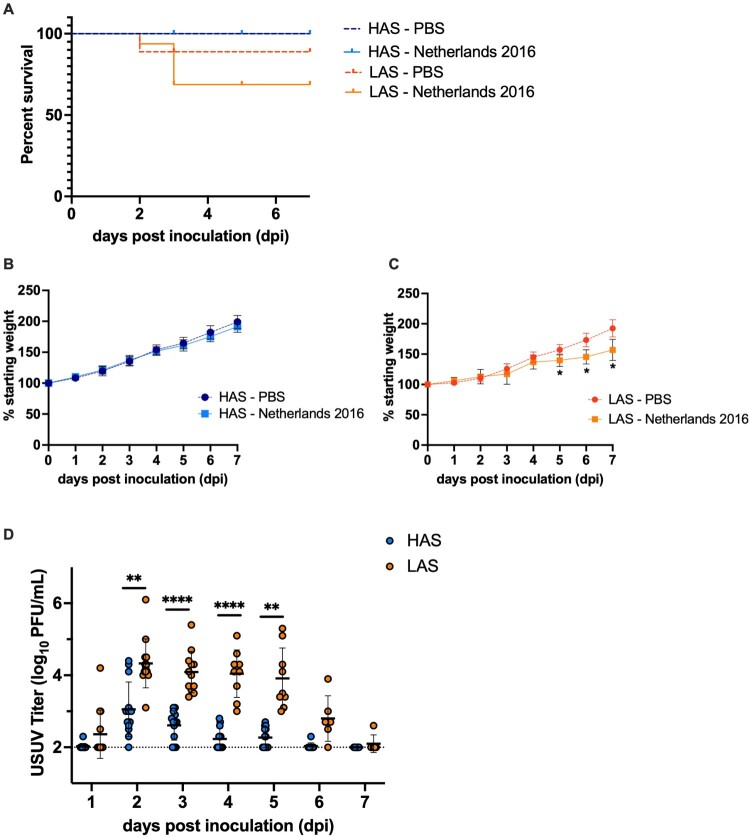
Mortality, morbidity, and viremia of HAS and LAS chickens inoculated with USUV. (A) Kaplan-Meier survival curve of chickens inoculated with 1500 PFU USUV or PBS. (B) Mean percentage of initial starting weight of HAS chickens following USUV inoculation. (C) Mean percentage of initial starting weight of LAS chickens following USUV inoculation. *p < 0.05. (D) Viremia of HAS and LAS chickens inoculated with 1500 PFU. Circles represent individual samples; lines represent mean; error bars represent standard deviation. The limit of detection is indicated by dashed line. **p < 0.01, ****p < 0.0001.

To establish a chicken model with more severe disease and higher serum titers, juvenile chickens from lines that had undergone long-term selection (47 generations) for high (HAS) or low (LAS) antibody production against sheep red blood cells [40,41] were subcutaneously inoculated with 1500 PFU of Netherlands 2016 or PBS as a control. While both the inoculated and control HAS groups had 100% survival, mortality in LAS groups was observed by dpi 2 (Figure 4(A)). There was 89% survival in the LAS control group and 69% survival in the LAS USUV inoculated group, with no significant differences in survival between groups. USUV-inoculated LAS chickens that did not survive exhibited disease signs including weight loss ≥15%, lethargy, and ruffled feathers.

Congruent with survivorship data, the mean weight of inoculated HAS chickens did not significantly differ from the control HAS group (Figure 4(B)). In contrast, the mean weight of LAS chickens inoculated with USUV was significantly lower than the control LAS group (Figure 4(C), *p *< 0.05) on dpi 5–7. Although these LAS juvenile chickens were growing and gaining weight, the data suggest that USUV infection diminished their rate of weight gain.

Viremia was measured daily for seven days following inoculation with USUV. All chickens developed viremia. Serum titers were significantly higher in LAS than HAS chickens on dpi 2–5 (Figure 4(D), *p *< 0.01, *p *< 0.0001). Both experimental groups reached peak viremia on dpi 2, with HAS chickens generating a mean 3.1 log_10_ PFU/mL titer and LAS chickens generating a mean 4.3 log_10_ PFU/mL titer.

### USUV dissemination and histopathology are significantly greater in LAS chickens

**Figure 5.**
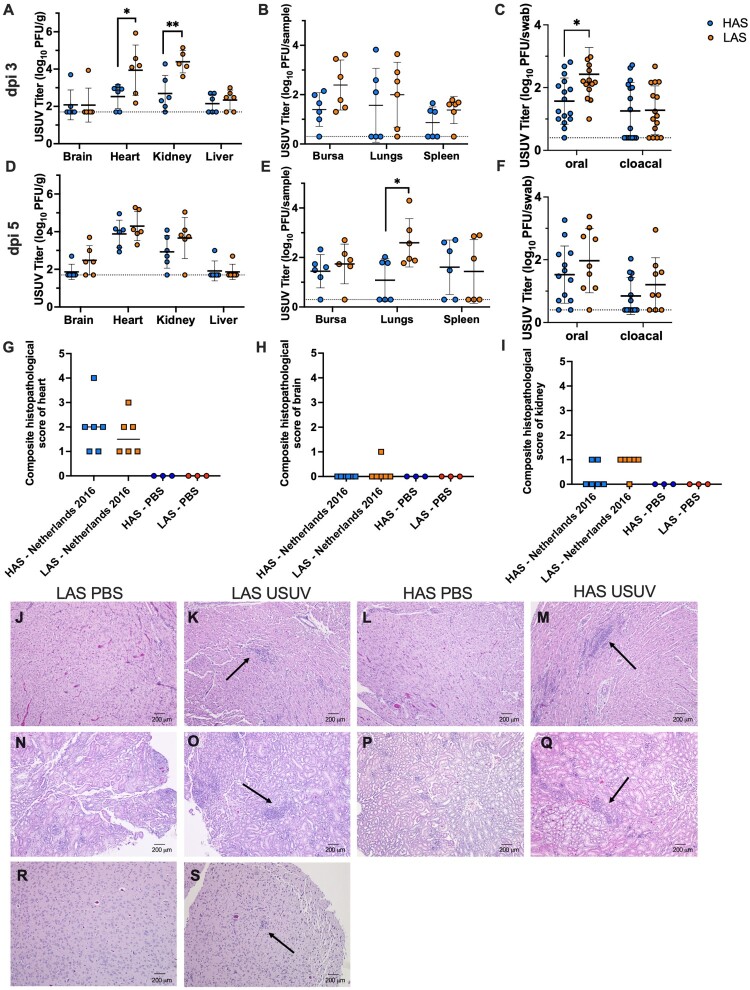
Evidence of viral dissemination, shedding, and histopathology in tissues of HAS and LAS chickens inoculated with USUV. (A) Viral titer in tissues collected on dpi 3. (B) Viral titer in tissues collected on dpi 3. (C) Viral titer in oral and cloacal swabs collected on dpi 3. (D) Viral titer in tissues collected on dpi 5. (E) Viral titer in tissues collected on dpi 5. (F) Viral titer in oral and cloacal swabs collected on dpi 5. Circles represent individual samples; lines represent mean; error bars represent standard deviation. The limit of detection is indicated by dashed line. *p < 0.05, **p < 0.01. (G) Composite score of heart tissue; line represents median. (H) Composite score of brain tissue; line represents median. (I) Composite score of kidney tissue; line represents median. (J) Representative image of heart tissue collected on dpi 5 from LAS chickens inoculated with PBS, with no inflammation (H&E stain). (K) Representative image of heart tissue collected on dpi 5 from LAS chickens inoculated with USUV, with inflammatory lesions and presence of heterophils and lymphocytes (arrow) (H&E stain). (L) Representative image of heart tissue collected on dpi 5 from HAS chickens inoculated with PBS, with no inflammation (H&E stain). (M) Representative image of heart tissue collected on dpi 5 from HAS chickens inoculated with USUV, with inflammatory lesions and presence of heterophils and lymphocytes (arrow) (H&E stain). (N) Representative image of kidney tissue collected on dpi 5 from LAS chickens inoculated with PBS, with no inflammation (H&E stain). (O) Representative image of kidney tissue collected on dpi 5 from LAS chickens inoculated with USUV, with heterophilic inflammatory foci (arrow) (H&E stain). (P) Representative image of kidney tissue collected on dpi 5 from HAS chickens inoculated with PBS, with no inflammation (H&E stain). (Q)Representative image of kidney tissue collected on dpi 5 from HAS chickens inoculated with USUV, with heterophilic inflammatory foci (arrow) (H&E stain). (R) Representative image of brain tissue collected on dpi 5 from LAS chickens inoculated with PBS, with no inflammation (H&E stain). (S) Image of brain tissue collected on dpi 5 from LAS chicken inoculated with USUV, with proliferation of glial cells in the neuropil (arrow) (H&E stain, scale bars = 200 µm).

Tissues were collected from six individuals on dpi 3 and dpi 5 to assess viral dissemination and pathology in each HAS and LAS experimental groups. Viral titers in the heart and kidney were significantly higher in LAS than HAS chickens on dpi 3 (Figure 5(A), *p *< 0.05, *p *< 0.01). Infectious virus was also isolated from brain, liver, bursa, lungs, and spleen of both HAS and LAS chickens (Figure 5(A, B)). Infectious virus was isolated from the panel of tissues collected on dpi 5 in both HAS and LAS chickens, with significantly higher USUV titer in the lungs of LAS chickens (Figure 5(D, E), *p*<0.05). Viral titers in the brain of LAS chickens were higher, though not significantly so, than HAS chicks on dpi 5 (Figure 5(D)).

To further evaluate the pathologic effect of USUV in HAS and LAS chickens, sections of tissues collected on dpi 5 were examined microscopically, with only heart, kidney, and brain showing evidence of disease. Tissues were semi-quantitively evaluated for the number of foci of inflammation; scores were summed for a composite pathology score. HAS and LAS chickens had similar median pathology scores in the heart (Figure 5(G)). Inflammation in the brain was observed in one LAS chicken (Figure 5(H)). HAS and LAS chickens had similar median pathologic scores in the kidney (Figure 5(I)). Heart tissue sections collected from both chicken lines were characterized by numerous inflammatory lesions, necrosis, lymphocytes, and heterophils, suggesting myocardial damage; whereas the sham-inoculated controls from each line did not exhibit signs of inflammation (Figures 5(J–M)). The kidney sections of both lines were characterized by inflammatory foci, including heterophils, with no signs of inflammation observed in the respective sham-inoculated controls (Figures 5(N–Q)). Proliferation of glial cells in the neuropil of one LAS chicken was observed and is suggestive of neuroinvasive disease (Figure 5(S)). There were no signs of inflammation observed in the respective sham-inoculated control (Figure 5(R)).

To assess viral shedding, oral and cloacal swabs were collected from all inoculated individuals on dpi 3 and dpi 5. USUV was isolated from oral swabs with significantly higher titers in LAS chickens than HAS chickens on dpi 3 (Figure 5(C), *p *< 0.05). USUV was also isolated from cloacal swabs of HAS and LAS chickens, with mean titers being similar on dpi 3 (Figure 5(C)). Infectious virus was isolated in oral and cloacal swabs of both groups on dpi 5 (Figure 5(F)). Together, these data suggest that USUV is more pathogenic in LAS compared to HAS chickens.

### HAS chickens generate a stronger neutralizing antibody response against USUV than LAS chickens

Serum was collected from HAS and LAS chickens on dpi 14 to evaluate rates of seroconversion using PRNT. There were significantly fewer LAS chickens that developed a neutralizing antibody response than HAS chickens (Table 1, Fisher’s exact test, *p *< 0.05). More than half of the HAS chickens neutralized USUV at titers of 40 or above. PRNTs were also performed on serum collected from HAS and LAS chickens on dpi 3, dpi 5, and dpi 7, but no sample reached the 90% reduction threshold. These data show that chickens selected to generate a higher antibody response to sheep red blood cells also develop a stronger neutralizing antibody response against USUV compared to those selected for low antibody response.

**Table 1.** Neutralizing antibody response in sera collected on dpi 14 from HAS and LAS chickens inoculated with USUV as determined by plaque reduction neutralization test at 90% threshold (PRNT_90_).

PRNT90 titer	% of subjects (*n*)		
	HAS	LAS	*p*-value
<20	10 (1)	66.67 (4)	0.0357
20	30 (3)	16.67 (1)	NS
0	40 (4)	16.67 (1)	NS
80	10 (1)	0 (0)	NS
320	10 (1)	0 (0)	NS

Percent (*n*) of exposed subjects reaching each titer is shown. NS denotes not significant.

### Viral clearance rate and infected cell removal are important for mitigating USUV viremia

**Figure 6.**
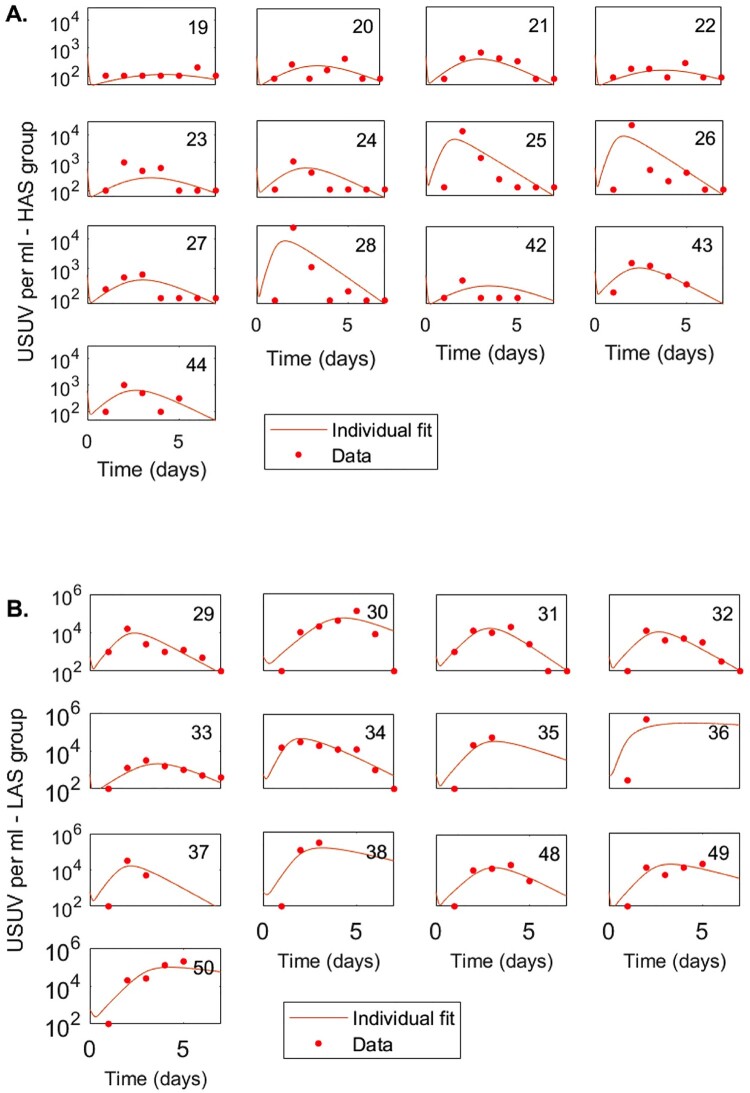
Mathematical modeling of HAS and LAS chickens inoculated with USUV. Kinetics of USUV in HAS and LAS chickens as given by model (Equation 1) versus data. A. Individual fits for HAS chickens B. Individual fits for LAS chickens. The parameters used in stimulation are given in Supplemental Table 3, k=0.97 per day, p=44.7 per infected cell per day, initial conditions are $T_{0}=10,000$ leukocytes/mL, $E_{0}=I_{0}=0$ leukocytes/mL, and $\log_{10}V_{0}=2.78$ PFU/ml. LAS chickens.

To determine whether differences in neutralizing antibody titers could explain differences in viremia in HAS and LAS chickens infected with USUV, a target cell-limited model with eclipse phase (Equation 1) was fitted to HAS and LAS virus titer data. Non-identifiability issues were avoided by assuming that the eclipse phase (1/k) and the virus production (p) are identical among the HAS and LAS populations, and fixing them at the combined populations estimate of 1 d and 44.7 virions per cell per day, respectively. The remaining parameters were assumed to be HAS- and LAS- specific and carried out as individual fits for each chicken within the HAS and LAS populations (see Supplemental Table 2, for the HAS and LAS average values). Data fitting showed good agreement with the experimentally determined virus load data, suggesting that peak serum titers occur when the number of newly infected cells no longer compensates for the loss of infected cells (Figure 6, see Supplemental Table 3 for corresponding individual fit data).

Differences in virus infectivity rates, infected cell loss, and virus removal were observed between HAS and LAS groups. The average estimate for the infectivity rate of individuals from the HAS population, $\text{β}=1.06\times 10^{-3}$ mL per virion per day, is 2.6 times higher than the average infectivity rate of the birds from the LAS population, $\text{β}=4.04\times 10^{-4}\;mL$ per virion per day, suggesting a lack of enhanced neutralizing antibody effects in the HAS group. The higher infectivity rate in the HAS cohort, however, is compensated by increased infected cell loss, δ=14.7 per day, 14.7-times larger than the infected cell loss in the LAS population, δ=1 per day. Additionally, the HAS group has enhanced virus clearance, c=8.5 per day, 2.7 times larger than the virus clearance in the LAS population, c=3.13 per day. This additional virus degradation in the HAS class is consistent with antibody-mediated neutralization.

The average basic reproduction number (or basic reproductive ratio), given by $R_{0}=\frac{\text{β}p}{c\delta }T_{0}$ was estimated to be $R_{0}=3.79$ for the HAS cohort, compared to $R_{0}=57.8$ for the LAS populations. This implies that the lower serum titers in HAS chickens reduces virus transmissibility by 15-fold.

## Discussion

This study revealed that USUV is pathogenic in juvenile chickens, with robust viral dissemination. Studies with three different lines of chickens (commercial ISA Brown, LAS, and HAS) indicated that juvenile chickens develop USUV viremia; USUV can be shed orally; and the heart is an important target organ for USUV replication and pathogenesis. Chickens inoculated with the South Africa 1959 strain had lower titers in serum, heart tissue, and oral swabs than chickens inoculated with contemporary strains (Figures 2(B) and 3(A–F)), suggesting that USUV may have evolved to increase replication in birds. Experimentally inoculated LAS chickens experienced morbidity that was not observed in either the HAS or commercial chickens. Additionally, LAS chickens generated significantly higher titers in serum, organ samples, and oral swabs (Figures 4 and 5), and a significantly lower virus-neutralizing immune response than did the HAS group. Altogether, juvenile chickens are an appropriate model for assessing USUV pathogenesis and transmission, in which weakened immune responses increase pathogenesis and transmission potential.

Histopathological analysis identified inflammation in heart tissue of USUV-inoculated chickens (Figures 3 and 5). Myocarditis and immune infiltrates in the heart have been reported in naturally infected wild birds in Europe, including the Eurasian blackbird, great grey owl, and house sparrow [21,47,48]. Inflammation was also observed in the brain of a single infected LAS chicken and in the kidney of both HAS and LAS groups, also consistent with USUV infected wild birds, as mentioned above [21,47,48]. While infectious virus was detected in the liver and spleen of LAS and HAS chickens, histopathological analysis did not reveal evidence of inflammation, whereas infiltrates of mononuclear cells and necrosis was observed in the spleen and liver of geese [29] chickens [30] and canaries [31] experimentally infected with USUV. Signs of inflammation following USUV infection were also observed in the heart and brain of experimentally inoculated canaries [31]. Thus, our results in juvenile chickens recapitulate many of the findings in experimental and natural USUV infections of various avian species. However, the degree of neuroinvasion was lower than expected based on reports of wild birds. While the LAS chickens did develop morbidity and neuropathology, they did not become as severely ill as described in some reports of wild birds. A further limitation of using a juvenile chicken model is that the poorly developed immune response of young chickens makes the model less useful for assessing therapeutics or prophylactic treatments to prevent disease.

The peak viremia generated in LAS chickens was 10^4^–10^5 ^PFU/mL. Thus, the LAS chicken line serves as a model that may be appropriate for determining the USUV viremia threshold for infectivity of *Culex* spp. mosquitos. Future studies to determine viremia threshold level for infectivity, coupled with data on wild bird susceptibility to USUV, can help determine which avian species are important for zoonotic viral maintenance and ultimately provide predictive capabilities for determining which bird populations are at risk for USUV emergence. Furthermore, bird-bird contact transmission has also been reported in experiments with WNV [49–51]. Our data showed that USUV is shed in oral and cloacal secretions of LAS chickens and, therefore, suggests the potential for USUV transmission between birds as well. However, there is currently no data on whether oral ingestion is a known transmission route for USUV.

When investigating a viral dose-dependent response, surprisingly, a higher viral inoculum dose corresponded to decreased viremia titers in chickens compared to those inoculated with a lower dose (Figure 2(B)). This phenomenon was also observed in three-week old chickens inoculated with SLEV [52], suggesting that high dose inoculum may be less effective at producing viremia in young chickens. Additionally, in wild birds experimentally inoculated with WNV, a higher inoculum dose did not significantly increase peak viremia titers but rather increased the proportion of infected birds [53–55]. Thus, our results indicate that in future avian experiments 1500 PFU is an appropriate dose for assessing USUV pathogenesis and transmission.

Results from our experiments with genetically distinct lines of chickens provide further insights into USUV-host interactions. The HAS chicken line has been well characterized to exhibit higher defensive responses, such as limited disease development, increased survivorship, and regular weight gain, when exposed to various pathogens including bacterial, viral, and parasitic [41]. Specifically, HAS chickens had 100% survivorship and developed a higher antibody response to Newcastle disease virus than their LAS counterparts. HAS chickens also developed fewer air sac lesions when challenged with *Mycoplasma gallisepticum* and had significantly higher weight gain when challenged with *Eimeria necatrix* than LAS chickens. HAS chickens were more resistant to splenomeglia virus and feather mites than their LAS counterparts. However, this high resistance was not comprehensive, as HAS chickens showed less resistance to *Escherichia coli* and *Staphylococcus aureus* infection than LAS chickens [41]. Genome sequencing and gene ontology analysis revealed three candidate genes responsible for driving the variance in humoral immune response between the HAS and LAS chicken lines: *MHC*, *SEMA5A*, and *TGFBR2* [42]. Differences in the *MHC* locus, which encodes for proteins involved in antigen presentation [56], likely explain in part the differential neutralizing antibody responses developed in the HAS and LAS chickens against USUV. Further characterization of the innate and non-neutralizing responses will be essential to understand the avian anti-USUV response, as the modelling results from our study indicate the HAS chickens have a greater rate of loss of infected cells and higher rate of viral clearance than the LAS chickens. This increased loss of infected cells in the HAS chickens may be due to antibody-dependent cellular cytotoxicity or complement mediated lysis. This is reflected in the sustained higher serum titers observed in the LAS chickens. This suggests that the HAS chickens are better able to control viral replication during acute infection. Whereas much of the current literature has explored the role humoral immunity plays in reducing WNV infections following repeat exposure in wild bird populations [49,57,58], there is limited knowledge on the interactions between the avian innate immune system and WNV. Newhouse and colleagues [59] elucidate some of the critical players in the avian innate immune response after experimentally infecting zebra finches (*Taeniopygia guttata*) with WNV. Future work on identifying the role of the innate immune system in USUV infection in birds will be critical for our understanding of this emerging zoonotic virus and the drivers of viral evolution.

Results from our studies have shed light on important characteristics of USUV infection, pathogenesis, and immune responses in a physiologically relevant model organism. The shared phylogenetic and ecological characteristics between USUV and WNV suggest that USUV has the potential to serve as an emerging threat. Many of the USUV outbreaks in Europe resulted in large die-off events of Eurasian blackbird (*T. merula*) populations. With the global distribution of related species, including the American robin (*T. migratorius*), which is a competent host for WNV [49,60], it is possible that USUV may continue to emerge. The data presented here and the development of a novel avian model provide vital tools for further evaluation of USUV pathogenesis and transmission in birds.
